# Bidirectional associations between parenting stress and child psychopathology: The moderating role of maternal affection

**DOI:** 10.1017/S0954579423001177

**Published:** 2023-09-29

**Authors:** Shou-Chun Chiang, Sunhye Bai

**Affiliations:** 1 Department of Human Development and Family Studies, The Pennsylvania State University, University Park, PA, USA; 2 The Ballmer Institute for Children’s Behavioral Health, University of Oregon, Eugene, OR, USA

**Keywords:** parenting stress, internalizing symptoms, externalizing symptoms, maternal affection, child development

## Abstract

Parenting stress and child psychopathology are closely linked in parent-child dyads, but how the bidirectional association varies across childhood and adolescence, and shifts depending on maternal affection are not well understood. Guided by the transactional model of development, this longitudinal, prospective study examined the bidirectional relations between parenting stress and child internalizing and externalizing problems and investigated the moderating role of maternal affection from childhood to adolescence. Participants were from the Future of Families and Child Wellbeing Study, a diverse, nationally representative sample of 2,143 caregiving mothers who completed assessments at children ages 5, 9, and 15. Using cross-lagged panel modeling, we found bidirectional effects between parenting stress and child internalizing and externalizing problems. However, additional multigroup analyses showed that bidirectional associations depend on the levels of maternal affection. In the high maternal affection group, parenting stress at age 5 predicted higher internalizing and externalizing problems at age 9, and reverse child-to-parent paths were found from age 9 to age 15. In contrast, only one cross-lagged path was found in the low maternal affection group. Findings suggest that maternal affection can heighten the transactional associations between parenting stress and child psychopathology.

## Introduction

Child internalizing and externalizing problems are linked to multiple aspects of later health and functioning. Identifying risk and protective factors associated with the development of internalizing and externalizing symptoms would improve prevention and intervention efforts to promote children’s well-being and reduce their risk for mental health problems in the future (Clark et al., 2010; Forbes et al., 2019). Parents are very influential to their children’s mental health (Bronfenbrenner, 1986; Dearing et al., 2006). At the same time, child mental health significantly influences parent well-being. This study extends past work on the bidirectional associations between parenting stress and child internalizing and externalizing problems from childhood to adolescence and examines the unique role of maternal affection in shaping these bidirectional associations in a sample of minoritized and socioeconomically at-risk sample of families.

### Conceptual framework

The transactional model of development emphasizes the bidirectional effects between parent and child characteristics over time, distinguishing parent-driven and child-driven effects on each member’s well-being (Sameroff, 2009). Further, the transactional model conceptualizes children as having active roles in families rather than being passive recipients of parents’ caregiving behaviors (Schermerhorn & Cummings, 2008). This is particularly true in adolescence, as children become more autonomous and exert greater influence on the family and the parent-child relationship (Bornstein & Putnick, 2018). The relationship between parenting stress and child well-being exemplifies this transactional model. Parenting stress refers to the negative psychological reactions to the demands of being a parent (Deater-Deckard, 1998). The stress in parenting is unique from other life stress (e.g., work stress) in that it directly impacts parent-child interactions and child development. Past studies have found that parenting stress, especially among mothers, is bidirectionally associated with child behavioral problems (Goodrum et al., 2021; Neece et al., 2012). However, most studies focus on the association between parenting stress and child externalizing problems (Gerstein & Poehlmann-Tynan, 2015; Mackler et al., 2015), and early to middle childhood (Cappa et al., 2011; Cherry et al., 2019; Stone et al., 2016). For instance, one study that assessed 404 parents and children oversampled for risk for externalizing problems at children ages 4, 5, 7, and 10 years revealed bidirectional associations between maternal parenting stress and child externalizing problems over the follow-up period (Mackler et al., 2015). Moreover, a study of 144 families with typically developing children and 93 families with identified developmentally delayed children indicated that child internalizing and externalizing problems were transactionally linked to parenting stress for both groups of children from ages 3 to 9 (Neece et al., 2012). Together, these findings support the transactional model in which parenting stress and child psychopathology, specifically externalizing problems, bidirectionally influence one another in early to middle childhood.

The longitudinal course of these bidirectional associations across childhood and adolescence is less studied. The transition from childhood to adolescence is a period of change for both the adolescent and the parent (McElhaney et al., 2009; McGue et al., 2005). Relatedly, adolescence marks a developmental period of heightened vulnerability for the onset of internalizing and externalizing problems due to biological and cognitive changes that take place during these years (Brieant et al., 2022; McLaughlin & King, 2015). Adolescents are more likely to express their needs and autonomy, and parents may experience role strains in caregiving and work-family conflicts (Nomaguchi, 2009; Scharlach, 2001). Also, parent-child relationships become more egalitarian, and conflicts become more frequent and intense in adolescence (Branje et al., 2008; Laursen & Collins, 2009), indicating that adolescents may play a greater role in their families and parents’ well-being. Thus, adolescents may be more sensitive to parenting stress, contributing to the transactional links between parents and adolescents. In contrast, children might be less aware of their parents’ stress and thus less likely to be impacted by parenting stress. However, few studies have examined whether transactional associations between parenting stress and child psychopathology differ across childhood and adolescence. The current study adds to this literature by examining these associations from children ages 5–9, and 9–15.

### Maternal affection as a relational moderator

Another limitation of previous research on the bidirectional association is that few studies consider the role of the affectionate relationships between parents and children. Maternal affection is defined by the extent to which mothers express or exhibit love, affection, and pride (e.g., responsiveness and supportiveness) toward their children (Aunola & Nurmi, 2004; Martin et al., 2015). There are two potential ways in which maternal affection may moderate the relationship between parenting stress and child psychopathology. First, maternal affection may buffer the negative influence of parenting stress on child mental health. Most past research supports this hypothesis; maternal affection promotes a close relationship between mothers and children, which is essential for healthy development (Buist et al., 2017; Ge et al., 2009; Laursen & Collins, 2009; McAdams et al., 2017). Mothers who are able to maintain high levels of affection, even when experiencing stress, may be able to protect their youth against the detrimental impact of parenting stress.

Alternatively, a close and tight-knit relationship that is reflected by high levels of maternal affection may enhance the negative effects of parenting stress on child well-being by promoting the transmission and co-regulation of negative emotions (Davies & Windle, 1997; Larson & Almeida, 1999). Indeed, members of close dyads, such as a parent and their offspring, often co-regulate their emotions, cognitions, and behaviors (Butler, 2011). High maternal affection generally reflects closeness and intimacy from mothers’ perspectives, suggesting that mothers have positive attitudes and evaluations toward their relationships with their children. Dyads characterized by high maternal affection may be more emotionally connected to their child, and show greater co-regulation of emotions with their child, in the day-to-day, which exacerbates the bidirectional association between parenting stress and child psychopathology from year to year. For example, children who reported being closer to their parents showed stronger same-day associations between stressors at school and parent-child conflict, indicating greater spillover of negative experiences from one context (i.e., school), to another (i.e., home) (Bai et al., 2017). The results imply that children who are close to their parents may allow their experiences at school to impact their interaction quality with their parents. Thus, a mother experiencing higher parenting stress may inadvertently transmit the distress to her closely bonded child, leading the child to also experience heightened levels of distress that over time contribute to greater levels of internalizing and externalizing problems.

A recent study further supports this hypothesis; parenting stress was positively related to adolescent internalizing and externalizing problems only when mothers showed a high level of affection toward the adolescents (Silinskas et al., 2020); however, this study only examined the unidirectional effects of parenting stress on child problems instead of the bidirectional associations over time. Therefore, the associations between parenting stress and child problems might be stronger for families with higher maternal affection compared to those with lower maternal affection, indicating a close, affective relationship would promote the transactional transmission of both positive and negative characteristics between parents and children.

Moreover, limited research has examined these processes in socioeconomically at-risk or racially and ethnically diverse families. Poverty poses great risk for parenting stress and child mental health problems, and significant socioeconomic, racial, and ethnic disparities exist in the prevalence of mental health problems (Ghandour et al., 2019; Masarik & Conger, 2017; Nomaguchi & House, 2013; Reiss, 2013). Minoritized and socioeconomically at-risk families face multiple uncontrollable challenges that impact the whole family, including minority and financial stress. These challenges likely exacerbate child psychopathology and burdens of child-rearing, and deprive resources that can facilitate parenting, The necessity of having to cope with racial bias, discrimination, or poverty impacts parents’ display and socialization of emotion, as well as children’s internalizing and externalizing problems (Dearing et al., 2006; Dunbar et al., 2017; Kim et al., 2011; Sirin et al., 2015). Thus, a closer examination of the moderating role of maternal affection in minoritized socioeconomically at-risk families can increase the understanding and generalizability of the transactional model of parenting stress. Specifically, the alternative conceptual framework on affect co-regulation suggests that close and affective parent-child relationship may accentuate the bidirectional links between parenting stress and child mental health during specific developmental periods. Based on recent literature (e.g., Bai et al., 2017; Silinskas et al., 2020) Drawing on the affective transmission framework, we hypothesized that the maternal affection might strengthen the bidirectional associations between parenting stress and child psychopathology. The current study adds to prior research by examining bidirectional links between parenting stress and child mental health from childhood to adolescence in an ethnically diverse sample of socioeconomically at-risk families with high and low levels of maternal affection.

### Current study

The present study utilized a prospective design to examine the bidirectional associations between parenting stress and child internalizing and externalizing problems in the context of maternal affection in a diverse, longitudinal sample of mothers and children at children ages 5, 9, and 15. The first goal was to investigate the bidirectional effects of parenting stress and child internalizing and externalizing problems from childhood to middle adolescence. Using cross-lagged longitudinal analyses, we tested the hypothesis that parenting stress and child problems may reciprocally influence each other, consistent with past studies (Cherry et al., 2019; Mackler et al., 2015). Moreover, we hypothesized that the transactional associations between parenting stress and child problems would be stronger in adolescence compared to childhood given that offspring behaviors impact their families and parents more during adolescence (e.g., Kochanova et al., 2022; Nomaguchi, 2009). The second goal was to test these bidirectional associations in families reporting high and low maternal affection at baseline. We hypothesized that high maternal affection may signal a dyadic relationship characterized by high co-regulation of behaviors and emotions – both positive and negative. We predicted that the bidirectional associations between parenting stress and child internalizing and externalizing symptoms would be stronger in dyads with high maternal affection. Additional sensitivity analyses were conducted to examine whether the findings held when controlling for sociodemographic characteristics (e.g., mother’s age, child race/ethnicity, number of children in the family).

## Methods

### Sample

Data are from the Future of Families and Child Wellbeing Study (FFCWS), a longitudinal study following a birth cohort of 4,898 children born in 15 states across 20 U.S. cities. FFCWS oversampled for nonmarital births between 1998 and 2000 with approximately three quarters of the children being born to unmarried parents. A stratified random sampling strategy was implemented in three stages by sampling cities, hospitals within cities, and births within hospitals. FFCWS recruited a diverse sample of children and parents who were at higher risk of poverty and other disadvantages (see Reichman et al., 2001 for more details). The FFCWS collected data from interviews with parents when the focal children were newly born (first wave) in the hospitals and age 1, and continued phone interviews and home visits when the child’s ages were three, five, nine, and 15. Given our emphasis on parenting stress across development, analytic sample in this study was limited to families in which the biological mother was the primary caregiver across all three waves. This enabled us to have consistency across the three waves while maximizing sample size. The analytical sample (*N* = 2,143, female = 48.7%) comprised of children who participated at age 5 (M_age_ = 5.09, SD_age_ = 0.19), 9 (M_age_ = 9.10, SD_age_ = 1.40) and 15 (M_age_ = 15.39, SD_age_ = 0.61) with their biological mothers. At age 15, youth identified as non-Hispanic African American (47.0%), Hispanic/Latino (21.5%), non-Hispanic White (16.6%), non-Hispanic Multi-racial (4.5%), and other (2.5%). Details of the analytical sample’s demographic characteristics are reported in the supplementary materials. Compared to the full FFCWS cohort, the analytical sample reported lower levels of maternal education (χ^2^(1) = 5.89, *p* < .001) and included more Non-Hispanic African-American children (χ^2^(1) = 2.87, *p* < .01). No significant differences in other study variables were found.

### Measures

#### Internalizing and externalizing problems

Child internalizing and externalizing problems were measured at the ages 5, 9, and 15 assessments using caregiver-reported items from the Child Behavior Checklist (CBCL) (Achenbach, 1991; Achenbach & Rescorla, 2001). The FFCSW administered developmentally appropriate items from the CBCL (Choi et al., 2018); thus, the total number of items was modified over time. At age 5, the internalizing problems included 16 items of the Anxious/Depressed and Withdrawn/Depressed scales (*α* = .72). Externalizing problems consisted of 30 items from the Aggressive and Delinquent/Rule-breaking Behaviors scales (*α* = .85). At age 9, 32 items of Anxious/Depressed, Withdrawn/Depressed, and Somatic Complaints scales made up the internalizing problems score (*α* = .84), and 35 items of Aggressive and Delinquent/Rule-breaking Behaviors comprised the externalizing problems score (*α* = .89). At age 15, 8 items from the Anxious/Depressed and Withdrawn/Depressed scales were included as internalizing problems (*α* = .80), and 20 items from the Aggressive and Delinquent/Rule-breaking Behaviors were included as externalizing problems (*α* = .88). Mothers rated their child’s behaviors on a 3-point scale from 0 (*not true*) to 2 (*often true*), and scores were averaged.

#### Parenting stress

Parenting stress was measured at the age 5, age 9, and age 15 assessments using four items drawn from the Child Development Supplement of the Panel Study of Income Dynamics (Hofferth et al., 1997), the Job Opportunities and Basic Skills Training Program (Hamilton et al., 2001), and Parenting Stress Index (Abidin & Brunner, 1995). The measure of parenting stress has been widely used in FFCWS studies (Cooper et al., 2009; Halpern-Meekin & Turney, 2016; Nomaguchi et al., 2017). Mothers were asked whether they agreed with the following four items on a 4-point scale from 1 (*strongly disagree*) to 4 (*strongly agree*): “Being a parent is harder than I thought it would be,” “I feel trapped by my responsibilities as a parent,” “I find that taking care of my child(ren) is much more work than pleasure,” and “I often feel tired, worn out, or exhausted from raising a family.” The four items were averaged to create a total score for parenting stress, and Cronbach’s α at each wave was .66, .66, and .69. Additional confirmatory factor analysis verified the validity of the parenting stress scale with all factor loadings greater than .05 as well as excellent model fit across waves (comparative fit index [CFI] = .99 ∼ 1.00; Tucker–Lewis index (TLI) = .98 ∼ .99; root mean square error of approximation [RMSEA] = .01 ∼ .03).

#### Maternal affection

Mothers’ affection toward their child, which consists of their expressions of love, affection, respect, admiration, and pride for their child, was measured using the Maternal Description of Child (MDoC) task during the age 5 FFCSW interview (Martin et al., 2015). Mothers were asked several open-ended questions, such as “*Now I*’*d like to get a general picture of CHILD. Can you tell me a little about him/her?*” and “*How do you feel about CHILD*’*s behavior when you are around other people?*” and their responses, audio recorded. Coders were instructed to consider the content of the number of positive statements, the level of description (i.e., specificity), and tone of voice as they rated the mother’s speech on a five-point scale: 1 = *Expresses 0–2 positive statements toward child and almost no positive tone* to 5 = *Nearly every statement is a positive one, tone is consistently positive and there is much specificity present in statements*. The Positive Affect subscale of MDoC had great convergent and predictive validity, as well as high interrater reliability (*r* = 0.84) (Martin et al., 2015).

##### Covariates.

Covariates included: child sex at birth, mother’s marital status (married vs. other types of relationships), mother’s education level (from 1 = less than high school to 4 = college and graduate degree), and household income-to-poverty ratio, which reflected the total household income to the federal poverty thresholds (the higher ratio indicated more financial resources) at age 5.

### Analytical strategy

Data analyses were performed using cross-lagged path models (CLPM) to test the longitudinal associations among parenting stress, internalizing problems, and externalizing problems using R software (R core team, 2017) with lavaan package (Rosseel, 2012). CLPM is commonly used in longitudinal study to explore the bidirectionality between two or more variables over time (Chiang & Bai, 2022; Lavner et al., 2017; Petren et al., 2021; Shaffer et al., 2013). To examine the first hypothesis, we utilized CLPM to examine cross-lagged coefficients across three age waves, as well as autoregressive coefficients of the same variables. Missing data were estimated using full information maximum likelihood (Enders & Bandalos, 2001), a robust estimation frequently used in panel studies to produce unbiased estimates and reduce potential effect of attrition (Graham, 2012; Little et al., 2014). Our three study variables were allowed to covary at each wave, accounting for the interdependence of the variables. We included child sex, marital status, mother’s education level, and household income-to-poverty ratio as covariates in the analytical models. We evaluated the model fit using the RMSEA, with values between 0.08 and 0.06 indicating acceptable fit and values < 0.06 indicating good fit; CFI and TLI, with values>0.90 indicating acceptable fit and values > 0.95 indicating good fit; and standardized root mean squared residual (SRMR), with values<0.05 indicating good fit (Bentler & Bonett, 1980; Hu & Bentler, 1999).

To investigate the second hypothesis, we conducted multigroup analyses to examine whether cross-lagged coefficients differed between dyads with lower and higher levels of maternal affection. Similar to past studies categorizing groups in multigroup analyses (e.g., Ding et al., 2017; Kim et al., 2018), we categorized families who scored 1 SD below the mean or less as low affection group (*N* = 301) and families who scored 1 SD above the mean or greater as high affection group (*N* = 278). Families within 1 SD from the mean (*N* = 834) were excluded in multigroup analyses. Families whose maternal affect score was between±1 SD from the mean were excluded from the multigroup analysis. Next, we compared the baseline model (i.e., freely estimated model of full sample) and a constrained model in which cross-lagged paths were constrained to be equal between the two groups. In addition, we also compared the baseline model to a constrained model in which autoregressive paths were constrained to be equal between the two groups. Given that chi-square tests are sensitive to sample size, we considered measurement invariance using difference in comparative fit index (ΔCFI). Thus, we determined model invariance when the ΔCFI was less than .01 (Chen, 2007; Cheung & Rensvold, 2002).

## Results

### Descriptive statistics

Means, standard deviations, and correlations of key study variables are shown in Table 1. Parenting stress was associated with greater internalizing problems and externalizing problems over the three-time points. In contrast, higher maternal affection was associated with fewer internalizing problems and externalizing problems. Child internalizing problems were positively associated with externalizing problems. We also conducted repeated measures ANOVAs to examine the overall differences between means of the key variables at the three-time points. The results indicated that parenting stress and externalizing problems were significantly higher at age 5 compared to age 15, and age 15 also significantly higher than age 9. Internalizing problems were significantly higher at age 5 and age 15 compared to age 9. In addition, we also examined whether key variables differed across families with high and low levels of maternal affection using *t* tests. The results showed that except for internalizing problems at age 15, parenting stress and externalizing problems at ages 5, 9, and 15, and internalizing problems at ages 5 and 9 were significantly higher in the low maternal affection group compared to high maternal affection group. A full description of ANOVAs and *t* tests analyses is available in Supplementary Material.

### Full sample model

**Figure 1.**
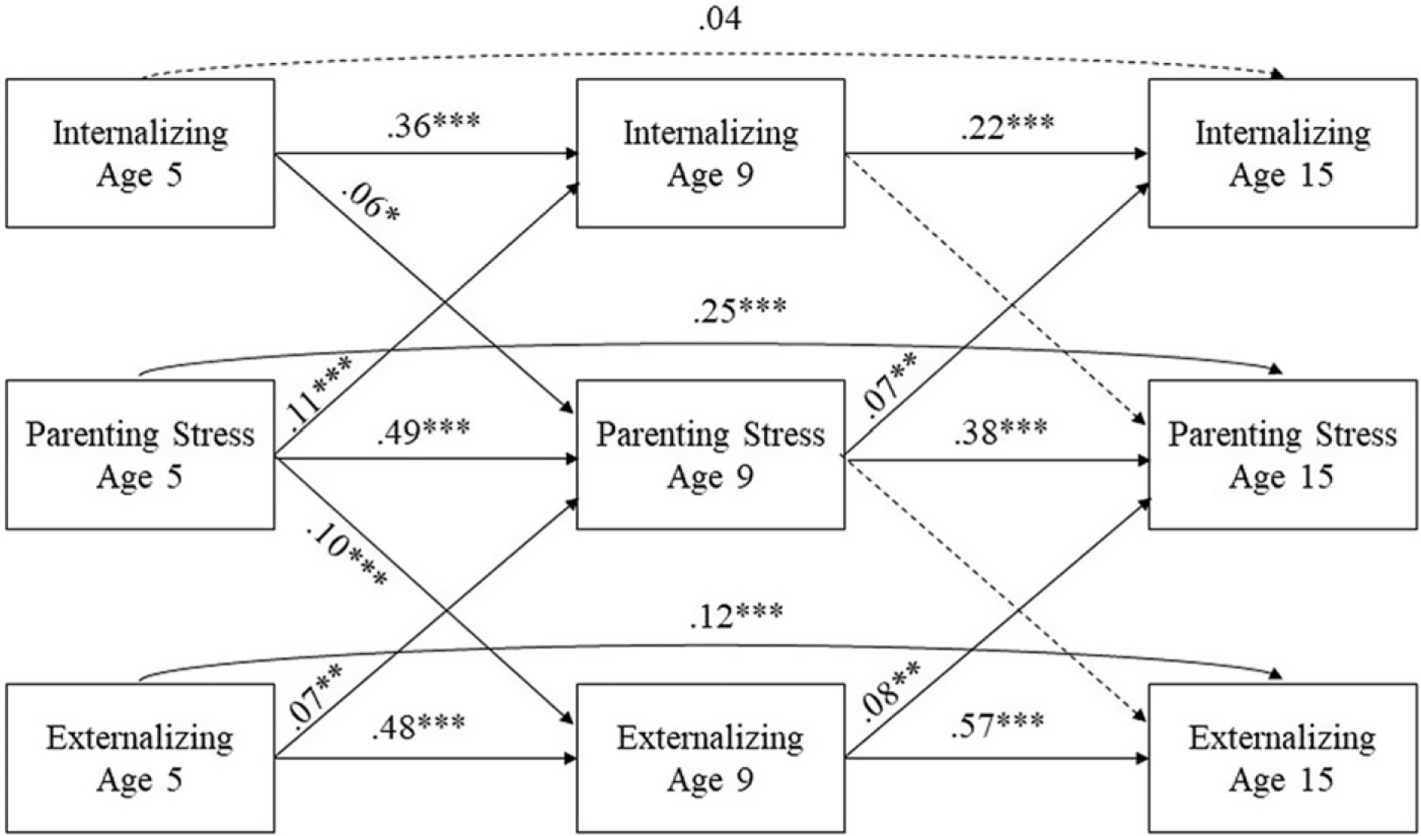
Cross-lagged conceptual model from age 5 to age 15. The model controlled for child sex, marital status, mother’s educational level, and household income-to-poverty as covariates. Internalizing: internalizing problems. Externalizing: externalizing problems. All concurrent variables were correlated. Study variables at the same wave were allowed to covary. *p < .05, **p < .01, ***p < .001.

We conducted CLPM analysis to examine the longitudinal associations between parenting stress, internalizing problems, and externalizing problems at ages 5, 9, and 15, as shown in Figure 1. The model fit the data well (χ^2^ (18) = 106.85, *p* < .001, CFI = .98, TLI = .97, RMSEA = 0.057, SRMR = 0.020). As shown in Table 2, parenting stress at age 5 significantly predicted greater internalizing problems (*β* = .11, *p* < .001) and externalizing problems (*β* = .10, *p* < .001) at age 9, and internalizing problems (*β* = .06, *p* < .05) and externalizing problems (*β* = .07, *p* < .01) at age 5 predicted greater parenting stress at age 9. Next, parenting stress at age 9 predicted greater internalizing problems at age 15 (*β* = .07, *p* < .01), and externalizing problems at age 9 predicted greater parenting stress at age 15 (*β* = .08, *p* < .01). However, internalizing problems at age 9 were not associated with parenting stress at age 15, and parenting stress at age 9 was not associated with externalizing problems at age 15. Finally, nine out of ten autoregressive coefficients were significant, indicating that parenting stress, internalizing problems, and externalizing problems were relatively stable over time.

### Multigroup analyses based on maternal affection

**Figure 2.**
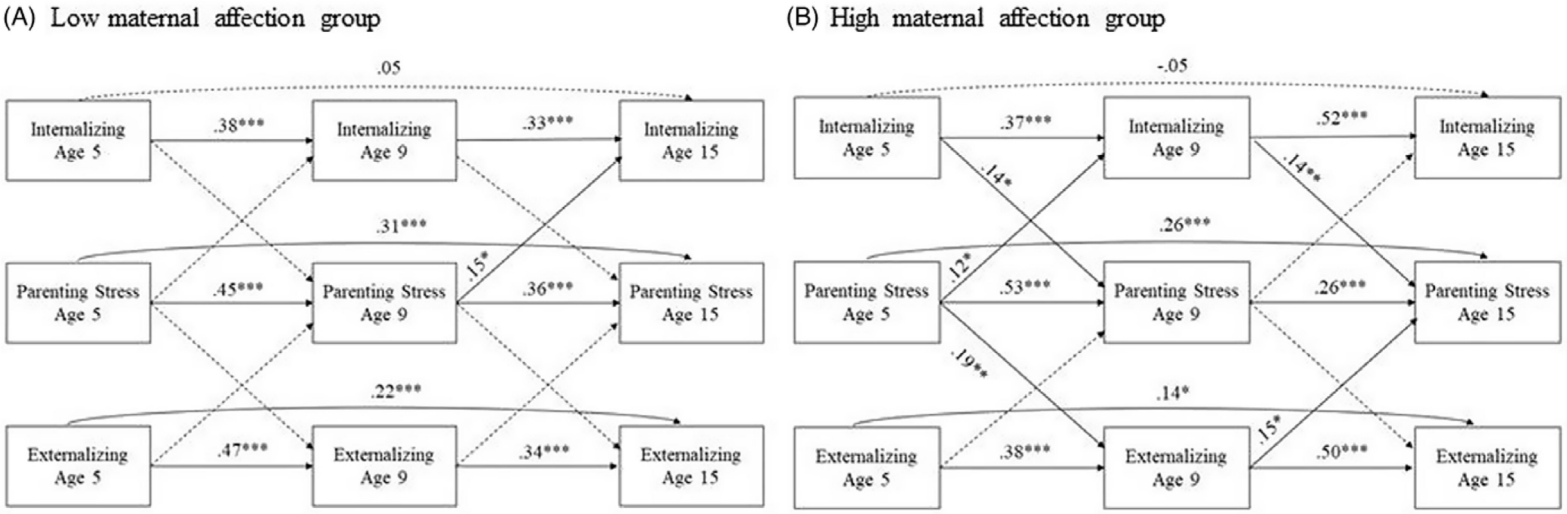
Cross-lagged models from age 5 to age 15 based on maternal affection. All coefficients were standardized. Study variables at the same wave were allowed to covary. The models controlled for child sex, marital status, mother’s educational level, and household income-to-poverty as covariates. Internalizing: internalizing problems. Externalizing: externalizing problems. Nonsignificant paths are indicated by dashed lines. *p < .05, **p < .01, ***p < .001.

We conducted multigroup analyses of the cross-lagged effects between parenting stress, internalizing problems, and externalizing problems for high and low levels of maternal affection as shown in Figure 2. The model fit the data well (χ^2^ (36) = 73.04, *p* < .001, CFI = .98, TLI = .98, RMSEA = 0.069, SRMR = 0.029). The tests model invariance showed that freely estimated model and cross-lagged constrained model were significantly different (ΔCFI = .03; χ^2^ (44) = 123.175, *p* < .001, CFI = .95, TLI = .96, RMSEA = 0.077, SRMR = 0.050), indicating cross-lagged paths were not equal between low and high maternal affection groups. However, the freely estimated model was invariant when we constrained the autoregressive paths (ΔCFI = .001), indicating no significant group differences in autoregressive paths. Thus, we examined cross-lagged coefficients for the two groups as shown in Table 3.

For the high maternal affection group, parenting stress at age 5 significantly predicted greater internalizing problems (*β* = .12, *p* < .05) and externalizing problems (*β* = .19, *p* < .01) at age 9. Internalizing problems at age 5 predicted greater parenting stress at age 9 (*β* = .14, *p* < .05). Externalizing problems at age 5 were not associated with parenting stress at age 9, and parenting stress at age 9 was associated with internalizing problems and externalizing problems at age 15. Furthermore, the significant associations between parenting stress and internalizing problems were not found for the low maternal affection group. Internalizing problems (*β* = .14, *p* < .05) and externalizing problems (*β* = .15, *p* < .05) at age 9 predicted greater parenting stress at age 15 for the high maternal affection group, whereas such associations were not found in low maternal affection group. One out of eight cross-lagged paths was significant in the low maternal affection group: parenting stress at age 9 predicted greater internalizing problems at age 15 (*β* = .15, *p* < .01).

### Sensitivity analyses

Finally, we conducted sensitivity analyses to examine whether the results remained when including additional sociodemographic covariates, including mother’s age, child race/ethnicity (non-Hispanic White; non-Hispanic Black; Hispanic; non-Hispanic other; non-Hispanic multi-racial), number of children in the family, immigrant status (i.e., whether the mother was born in the U.S.), and maternal depression. Results indicated that the directions and significance of path coefficients did not substantially change after the inclusion. However, model fit index based on alternative models with child race/ethnicity was not acceptable (χ^2^ = 427.50, *p* < .001; CFI = .817; RMSEA = 0.984; SRMR = 0.082), precluding our interpretations of path coefficients controlling for child race/ethnicity.

## Discussion

The current study examined bidirectional associations between parenting stress and child internalizing and externalizing problems from ages 5 to 15, among racially and ethnically diverse socioeconomically at-risk families reporting high and low levels of maternal affection in the Future of Families Child Wellbeing Study. Bidirectional effects of parenting stress and child internalizing and externalizing problems were found from ages 5 to 9, and to a more limited extent, ages 9 to 15. Next, results showed that bidirectional associations between parents and children were different based on levels of maternal affection. Bidirectional associations between parenting stress and child internalizing and externalizing problems were more prevalent in the high maternal affection group and less so, in the low maternal affection group. Moreover, the high maternal affection group demonstrated that parenting stress predicted child internalizing and externalizing problems from children ages 5 to 9, whereas child internalizing and externalizing problems predicted parenting stress from children ages 9 to 15. The findings underscore the importance of considering the transactional processes in close parent-child relationships characterized by maternal affection.

### Bidirectional associations between parenting stress and child internalizing and externalizing problems over time

Our findings indicated that prospective associations between parenting stress and child internalizing and externalizing problems were mostly bidirectional in the full sample. Consistent with findings during infancy and childhood (Sameroff, 2009; Stone et al., 2016), this study found that parenting stress at age 5 predicted child internalizing and externalizing problems at age 9; likewise, child internalizing and externalizing problems predicted parenting stress. These results support the theory that both parents and children mutually affect each other’s health and development (Cox & Paley, 2003; James et al., 2021; Sameroff, 2009). Moreover, our study revealed that the bidirectional associations between parents and children were more evident in childhood compared to adolescence. From age 9 to 15, parenting stress only predicted later internalizing problems, while externalizing problems predicted later parenting stress from age 9 to 15. It is possible that the significant biological and psychological changes that children undergo during the transition to adolescence (Eccles et al., 2018; Graber & Brooks-Gunn, 1996), including the development of non-familial bonds with peers, increased levels of behavioral autonomy, and less supervised time with parents, would reduce the prospective impact of parenting stress on later externalizing problems. Still, parenting stress predicted later internalizing symptoms, perhaps reflecting past findings that stress in the family environment during childhood poses significant risk for adolescent depression via increased youth sensitivity to stressors (Duggal et al., 2001; Rao & Chen, 2009; St Clair et al., 2015).

### Multigroup analyses between high and low maternal affection

The findings of multigroup analyses support our hypotheses that maternal affection, indicating closeness in the dyad, could facilitate the transactional processes underlying the bidirectional associations between parenting stress and child internalizing and externalizing problems. In the high maternal affection group, parenting stress predicted more child internalizing and externalizing problems, and internalizing problems predicted greater parenting stress from age 5 to 9. Thus, in middle childhood, parent-driven effects on child problems were more pronounced than child-driven effects on parenting stress. For this same group, internalizing and externalizing problems predicted greater parenting stress from childhood to adolescence; parenting stress no longer predicted adolescent psychopathology. In contrast, only one unidirectional effect was found for the low maternal affection group. Parenting stress predicted greater internalizing problems from middle childhood to middle adolescence; no other significant paths were found. These provide partial support for the bidirectional associations between parenting stress and child psychopathology in high maternal affection dyads; links between parenting stress and child psychopathology in low maternal affect are minimal (Pettit & Arsiwalla, 2008). Specifically, transactional effects between parenting stress and child psychopathology were more evident for families with higher levels of maternal affection because high maternal affection in early childhood might yield more opportunities to facilitate the transmission of negative affect and distress. Furthermore, the families in the sample were predominantly African American or Hispanic, and socioeconomically at-risk. Youth in these groups have a higher prevalence of depression and behavioral problems, and parents with greater parenting stress (Ghandour et al., 2019; Nomaguchi & House, 2013; Reiss, 2013). These families also face significant extrafamilial stress, such as financial and minority stress, that can exacerbate parenting stress and mental health problems. These extrafamilial stressors impact all members of the family, those characterized by high levels of affection might be especially prone to the transmission of negative emotion.

Even in these close relationships, however, the effects of parenting stress on later internalizing and externalizing problems become weaker as youth transition from childhood to adolescence. Rather, children’s behaviors contributed to parenting stress from age 9 to 15, perhaps reflecting greater influence on the family system gained by youth during this developmental period. The lack of significant associations found in the low maternal affection group may be due to the parents and children not having the opportunity to display their challenges to the other membersof the dyad (Chan et al., 2015). While both members may experience poor emotional well-being, their difficulties may be less linked because there are fewer opportunities for transactional exchanges of emotions, behaviors, and cognitions to unfold.

Together, our findings underscore the role of maternal affection in the bidirectional associations between parenting stress and child internalizing and externalizing problems in a diverse and socioeconomically at-risk sample of mother-child dyads. Existing literature has emphasized the bidirectional influences between parenting stress and child psychopathology (Burke et al., 2008); however, few considered the potential function of maternal affection in shaping these bidirectional associations in racially, ethnically, and socioeconomically marginalized groups. Our results supported our hypotheses that a certain level of intimacy and affection is needed for parents and children to bidirectionally influence each other, even in ways that are less desirable for parent and child mental health. Past research has indicated that early experience of affectionate and warm interactions between parents and children has lasting effects on later development of children (Del Barrio et al., 2016; Lamb & Lewis, 2011 Waters et al., 2000). Our findings found that maternal affection at age 5 would not only influence child development but also moderate the bidirectional associations between parents and children from childhood to middle adolescence. Thus, early maternal affection may serve as a critical contextual factor that shapes the transactional links between parenting stress and child psychopathology, suggesting that early characteristics of the parent-child relationship have long-term implications for the health of the family and children.

Study findings have important implications for clinical practice, pending replication. First, the bidirectional linkages between parenting stress and child mental health outcomes highlight the importance of supporting all members of the family, in addition to the identified patient. Second, findings emphasize the importance of recognizing that there may be multiple facets to maternal affection and that some facets may be protective, whereas others, detrimental to individuals’ emotional well-being. For example, high levels of enmeshment, defined by family systems theorists as a tendency for two members of a dyad to have boundaries that are overly diffuse and permeable (Coe et al., 2018; Minuchin, 1985), may underlie maternal affection, and drive the bidirectional association between parenting stress and child outcomes. Future clinical research can further clarify our understanding of the multiple facets of maternal affection, and examine contextual factors that shape their generally protective effects on child well-being.

Despite several strengths of the study, including the diverse, large-scale sample of families, robust effects after controlling multiple confounding variables, and spanning across childhood and adolescence, several limitations should be noted. First, the study did not include fathers’ parenting stress due to most caregivers of the FFCWS being mothers. Although past studies on parenting stress showed that fathers’ parenting stress had small or no impact on children’s well-being among normative and risky populations (Rodriguez et al., 2019; Ward & Lee, 2020), fathers’ effect remains an important factor of family functioning and child development (Cabrera et al., 2018; Li & Meier, 2017). Thus, future research should include parenting stress of fathers as well as father reports of child psychopathology to investigate the bidirectional effects between parents and between mother-child and father-child processes. Secondly, we were not able to include all racial/ethnic covariates due to poor model fit. Although our findings represented average effects across the diverse racial/ethnic families, exploring whether these effects are heterogeneous across specific groups is an important direction for future research. Next, most constructs were assessed by self-report questionnaires and only maternal affection was ascertained by independent raters. Applying more fine-grained designs (e.g., ecological momentary assessment) and home observational studies would reduce recall bias and provide real-time evidence (day-to-day, moment-to-moment) of parent-child interactions that contribute to child development unfolding over the long term (Chiang et al., 2023; Repetti et al., 2015; Russell & Gajos, 2020). Moreover, the associations between parenting stress and child problems may be inflated by same-reporter bias (Valdes et al., 2016). Evaluating the bidirectional associations from multiple reporters is a crucial next step for future studies. In addition, parenting stress was assessed by only four items at each wave, which might posit concerns about the internal consistency. Future research should adopt more validated measures of parenting stress to ensure the scale reliability. Also, we focused on the biological mothers who consistently participated in the FFCWS across three waves, which might limit generalizability for families with non-biological mothers and fathers as primary caregivers. Despite the importance of early experience of maternal affection as demonstrated in the present study, we acknowledged that such characteristics of parent-child relationship may change over the course of child development. However, maternal affection was only assessed at age 5 in the FFCWS, limiting our ability to explore the role of maternal affection at different ages. Thus, additional studies are needed to investigate how maternal affection may vary and moderate the transactional links between parent-child characteristics. Finally, although we examined diverse sample in the U.S., parenting stress and child psychopathology may differ by unique cultural values and other characteristics of the macrosystem. Future research should examine parenting stress and child mental health in the context of specific cultural values, varying levels of economic resources, and socio-political climates, as these contextual factors heavily influence the health of families and youth.

In conclusion, the current study makes a unique contribution to the understanding of bidirectional associations between parenting stress and child psychopathology at different levels of maternal affection. The findings demonstrate that maternal affection is an important family characteristic that shapes the prospective associations between parenting stress and child internalizing and externalizing problems. Prevention and intervention efforts should consider both the advantages and potential disadvantages that a highly intimate parent-child relationship may have on parents’ and children’s well-being and development. The findings call for more comprehensive evaluations of parent-child relationships and the relevant developmental outcomes.

## Supplementary Material

1

## Figures and Tables

**Table 1.** Descriptive statistics and correlations at ages 5, 9, and 15

	1.	2.	3.	4.	5.	6.	7.	8.	9.	10.
1. Internalizing Problems, age 5	1									
2. Internalizing Problems, age 9	.34***	1								
3. Internalizing Problems, age 15	.21***	.34***	1							
4. Externalizing Problems, age 5	.44***	.20***	.22**	1						
5. Externalizing Problems, age 9	.23***	.57***	.33**	.49***	1					
6. Externalizing Problems, age 15	.15***	.22***	.51**	.40***	.52***	1				
7. Parenting Stress, age 5	.19***	.17***	.17**	.23***	.20***	.15***	1			
8. Parenting Stress, age 9	.16***	.23***	.19**	.21***	.30***	.20***	.52***	1		
9. Parenting Stress, age 15	.15***	.20***	.29**	.19***	.26***	.32***	.50***	.55***	1	
10. Maternal Affection, age 5	−.18***	−.15***	−.06*	−.28***	−.21***	−.20***	−.17***	−.16***	−.14***	1
*Mean*	0.27	0.18	0.26	0.38	0.19	0.22	2.18	2.03	2.07	2.96
Range	0–3	0–3	0–3	0–3	0–3	0–3	1–4	1–4	1–4	1–4
*SD*	0.22	0.20	0.31	0.24	0.17	0.25	0.67	0.69	0.71	0.79

* *p* < .05.

** *p* < .01.

*** *p* < .001.

**Table 2.** Full sample cross-lagged model

	*β*	*SE*	95% CI
*Cross-lagged coefficients*			
Parenting stress_5_ → Internalizing problems_9_	.11***	0.01	[0.06, 0.15]
Parenting stress_5_ → Externalizing problems_9_	.10***	0.01	[0.06, 0.15]
Internalizing problems_5_ → Parenting stress_9_	.06*	0.07	[0.01, 0.11]
Externalizing problems_5_ → Parenting stress_9_	.07**	0.06	[0.03, 0.12]
Parenting stress_9_ → Internalizing problems_15_	.07**	0.01	[0.03, 0.12]
Parenting stress_9_ → Externalizing problems_15_	.04	0.01	[−0.01, 0.10]
Internalizing problems_9_ →Parenting stress_15_	.02	0.10	[−0.02, 0.07]
Externalizing problems_9_ →Parenting stress_15_	.08**	0.06	[0.03, 0.13]
*Autoregressive coefficients*			
Parenting stress_5_ → Parenting stress_9_	.49***	0.02	[0.45, 0.53]
Parenting stress_9_ → Parenting stress_15_	.38***	0.02	[0.34, 0.44]
Parenting stress_5_ → Parenting stress_15_	.25***	0.03	[0.21, 0.30]
Internalizing problems_5_ →Internalizing problems_9_	.36***	0.02	[0.31, 0.40]
Internalizing problems_9_ →Internalizing problems_15_	.30***	0.05	[0.25, 0.35]
Internalizing problems_5_ →Internalizing problems_15_	.04	0.02	[−0.01, 0.08]
Externalizing problems_5_ →Externalizing problems_9_	.47***	0.02	[0.43, 0.51]
Externalizing problems_9_ →Externalizing problems_15_	.47***	0.04	[0.42, 0.52]
Externalizing problems_5_ →Externalizing problems_15_	.13***	0.02	[0.09, 0.17]
*Concurrent correlations*			
Internalizing problems_5_ ↔ Parenting stress_5_	.18***	0.02	[0.13, 0.23]
Parenting stress_5_ ↔ Externalizing problems_5_	.24***	0.02	[0.19, 0.28]
Internalizing problems_5_ ↔ Externalizing problems_5_	.43***	0.02	[0.39, 0.47]
Internalizing problems_9_ ↔ Parenting stress_9_	.18***	0.02	[0.13, 0.22]
Parenting stress_9_ ↔ Externalizing problems_9_	.18***	0.02	[0.13, 0.23]
Internalizing problems_9_ ↔ Externalizing problems_9_	.55***	0.02	[0.51, 0.58]
Internalizing problems_15_ ↔ Parenting stress_15_	.19***	0.01	[0.15, 0.24]
Parenting stress_15_ ↔ Externalizing problems_15_	.25***	0.01	[0.20, 0.29]
Internalizing problems_15_ ↔ Externalizing problems_15_	.46***	0.01	[0.42, 0.49]

*β* = standardized coefficient; R^2^ = parenting stress_15_; *SE* = standard error.

The subscripts refer to the assessment age. The model controlled for child sex, marital status, mother’s educational level, and household income-to-poverty as covariates.

(.35); Internalizing problems_15_ (.18); Externalizing problems_15_ (.28).

* *p* < .05.

** *p* < .01.

*** *p* < .001.

**Table 3.** Cross-lagged multigroup models for low and high maternal affection groups

	Low affection			High affection		
*Cross-lagged coefficients*	*β*	*SE*	95% CI	*β*	*SE*	95% CI

Parenting stress_5_ → Internalizing problems_9_	.01	0.02	[−0.11, 0.13]	.12*	0.01	[0.01, 0.24]

Parenting stress_5_ → Externalizing problems_9_	.06	0.02	[−0.06, 0.18]	.19**	0.01	[0.08, 0.30]

Internalizing problems_5_ → Parenting stress_9_	.12	0.19	[−0.02, 0.25]	.14*	0.21	[0.03, 0.26]

Externalizing problems_5_ → Parenting stress_9_	.11	0.15	[−0.02, 0.24]	.01	0.20	[−0.11, 0.13]

Parenting stress_9_ → Internalizing problems_15_	.15*	0.02	[0.02, 0.28]	−.08	0.03	[−0.20, 0.04]

Parenting stress_9_ → Externalizing problems_15_	.03	0.02	[−0.11, 0.16]	.00	0.02	[−0.12, 0.11]

Internalizing problems_9_ →Parenting stress_15_	.09	0.28	[−0.06, 0.24]	.14*	0.33	[0.01, 0.27]

Externalizing problems_9_ →Parenting stress_15_	−.07	0.28	[−0.22, 0.08]	.15*	0.31	[0.02, 0.28]

*β* = standardized coefficient; R^2^ = parenting stress_15_; *SE* = standard error.

The subscripts refer to the assessment age point. The model controlled for child sex, marital status, mother’s educational level, and household income-to-poverty as covariates. (Low: .33, High: .37); Internalizing problems_15_ (Low: .18, High: .37); Externalizing problems_15_ (Low: .18, High: .26).

* *p* < .05.

** *p* < .01.

*** *p* < .001.
