# Supplementary material for: Bidirectional associations between parenting stress and child psychopathology: The moderating role of maternal affection
Source: Dev Psychopathol. Author manuscript; Available in PMC 2025 Mar 29. (PMC10978553; doi:10.1017/S0954579423001177)
Supplement: 1 [file NIHMS1928278-supplement-1.docx]

To examine patterns of parenting stress, internalizing problems, and externalizing problems across three waves, we conducted a series of repeated measures analysis of variance (ANOVA). The results showed that parenting stress significantly differed across waves, F(2, 2,121) = 58.27, p < .001, with age 5 significantly higher than age 15, and age 15 significantly higher than age 9. Similarly, externalizing problems significantly differed across waves, F(2, 1,811) = 635.34, p < .001, with age 5 significantly higher than age 15, and age 15 significantly higher than age 9. Last, internalizing problems significantly differed across waves, F(2, 1,803) = 149.30, p < .001, with age 5 and age 15 significantly higher than age 9. We also conducted a series of independent samples t-tests to examine whether our main variables vary by groups of high and low maternal affection. The results showed that, compared to high maternal affection families, low maternal affection families had higher parenting stress at age 5 (*t* test of difference: *p* < .001), age 9 (*t* test of difference: *p* < .001), and age 15 (*t* test of difference: *p* < .001), higher internalizing problems at age 5 (*t* test of difference: *p* < .001), age 9 (*t* test of difference: *p* < .001), and higher externalizing problems at age 5 (*t* test of difference: *p* < .001), age 9 (*t* test of difference: *p* < .001), and age 15 (*t* test of difference: *p* < .001). Internalizing problems at age 15 were not significantly different across two groups (*t* test of difference: *p* = .075).

**S1**

*Results of Covariates in Full Sample Cross-Lagged Model*

|  | Parenting stress_15_ | | Internalizing problems_15_ | | Externalizing problems_15_ | |
| --- | --- | --- | --- | --- | --- | --- |
|  | *β* | *SE* | *β* | *SE* | *β* | *SE* |
| Sex | -.04* | 0.03 | .07* | 0.01 | .02 | 0.01 |
| Marital status | -.03 | 0.01 | -.04 | 0.01 | -.06* | 0.01 |
| Education level | -.01 | 0.01 | .04 | 0.01 | .03 | 0.01 |
| Poverty ratio | .03 | 0.01 | .05* | 0.01 | .06* | 0.01 |

Note. Sex: was coded as 1 (female) and 0 (male); Marital status was coded as 1 (married) and 0 (other types of relationships).

* *p* < .05, ** *p* < .01, *** *p* < .001

Lavaan Syntax for Models

Full sample Cross-Lagged Model

model <- '

# direct effect

internalw2 ~ internalw1 + externalw1+ psw1

externalw2 ~ internalw1 + externalw1+ psw1

psw2 ~ internalw1 + externalw1+ psw1

internalw3 ~ internalw2 + externalw2+ psw2

externalw3 ~ internalw2 + externalw2+ psw2

psw3 ~ internalw2 + externalw2+ psw2

#autoregression

psw3 ~ psw1

internalw3 ~ internalw1

externalw3 ~ externalw1

#intercepts

internalw1 ~ 1

internalw2 ~ 1

internalw3 ~ 1

externalw1 ~ 1

externalw2 ~ 1

externalw3 ~ 1

psw1 ~ 1

psw2 ~ 1

psw3 ~ 1

'

fit <- sem(model, data = data)

Multigroup Cross-Lagged Model

model <- '

# direct effect

internalw2 ~ internalw1 + externalw1+ psw1

externalw2 ~ internalw1 + externalw1+ psw1

psw2 ~ internalw1 + externalw1+ psw1

internalw3 ~ internalw2 + externalw2+ psw2

externalw3 ~ internalw2 + externalw2+ psw2

psw3 ~ internalw2 + externalw2+ psw2

#auto

psw3 ~ psw1

internalw3 ~ internalw1

externalw3 ~ externalw1

#intercepts

internalw1 ~ 1

internalw2 ~ 1

internalw3 ~ 1

externalw1 ~ 1

externalw2 ~ 1

externalw3 ~ 1

psw1 ~ 1

psw2 ~ 1

psw3 ~ 1

'

fit <- sem(model, data = data, group = “maternal_affection_group”,missing=’fiml’)
